# Does doctors’ workload impact supervision and ward activities of final-year students? A prospective study

**DOI:** 10.1186/1472-6920-12-24

**Published:** 2012-06-11

**Authors:** Nora Celebi, Rodoula Tsouraki, Corinna Engel, Friederike Holderried, Reimer Riessen, Peter Weyrich

**Affiliations:** 1 Department of Internal Medicine, Division of Diabetes, Endocrinology, Angiology, Nephrology and Clinical Chemistry, University Hospital of Tübingen, Tübingen, Germany; 2 University of Tübingen, Medical School, Tübingen, Germany; 3 Institute for Medical Biometry, University of Tübingen, Tübingen, Germany; 4 Department of the Dean of Student Affairs, University of Tübingen, Tübingen, Germany; 5 Department of Internal Medicine, Medical Intensive Care Unit, University Hospital of Tübingen, Tübingen, Germany

## Abstract

****Background**:**

Hospital doctors face constantly increasing workloads. Besides caring for patients, their duties also comprise the education of future colleagues. The aim of this study was to objectively investigate whether the workload arising from increased patient care interferes with student supervision and is associated with more non-medical activities of final-year medical students.

****Methods**:**

A total of 54 final-year students were asked to keep a diary of their daily activities over a three-week period at the beginning of their internship in Internal Medicine. Students categorized their activities – both medical and non-medical - according to whether they had: (1) only watched, (2) assisted the ward resident, (3) performed the activity themselves under supervision of the ward resident, or (4) performed the activity without supervision. The activities reported on a particular day were matched with a ward specific workload-index derived from the hospital information system, including the number of patients treated on the corresponding ward on that day, a correction factor according to the patient comorbidity complexity level (PCCL), and the number of admissions and discharges. Both students and ward residents were blinded to the study question.

****Results**:**

A total of 32 diaries (59 %, 442 recorded working days) were handed back. Overall, the students reported 1.2 ± 1.3 supervised, 1.8 ±1.6 medical and 3.6 ± 1.7 non-medical activities per day. The more supervised activities were reported, the more the number of reported medical activities increased (p < .0001). No relationship between the ward specific workload and number of medical activities could be shown.

****Conclusions**:**

There was a significant association between ward doctors’ supervision of students and the number of medical activities performed by medical students. The workload had no significant effect on supervision or the number of medical or non-medical activities of final-year students.

## **Background**

Over the past decade, an aging population and increasing economical restraints have led to considerable efforts to optimize health care systems across the world [1,2]. For most doctors, these changes have entailed having to care for more patients with more comorbidities in a shorter space of time, resulting in a considerable increase in workload in regard to patient care [3,4].

Beside patient care, providing medical education for future colleagues represents a further important aspect of most doctors’ daily work. When it comes to helping medical students master complex medical tasks, supervision, including the provision of adequate feedback, remains the gold standard [5,6]. In internships, students are supposed to learn from their doctors on ward [7]. However, many final-year medical students complain about the lack of time of the ward doctors available for supervision and even about being rather exploited for non-medical activities (activities that could be delegated to nurses or non-medical staff) in order to help doctors cope with an increasing workload in patient care [7,8].

In focus group analysis of van der Zwet et al. and Dornan et al., the students stated that the great advantage of workplace learning was “learning by doing” [9,10]. However, the students are consequently torn between doing something themselves and delivering safe patient care [11]. In a focus group analysis by de Feijter et al., the relationship to their supervisor was essential when it came to taking responsibilities, building up trust and asking for supervision [11].

Studies that focus on the supervision, feedback and assessment of medical students during clerkships generally show that the situation is not optimal. A survey from the Netherlands by Daelsmans et al. among 81 medical students reported that the supervision rate was less than 35 % in almost all competencies examined, and that the rate of direct observation was even lower [12]. In a study by Howley et al. among 345 medical students in the U.S., 51 % of the students reported that they had never been observed during history taking (81 % for a complete physical examination) by faculty staff [13].

While history taking and physical examination are usually performed from day one of the first clerkship, some other medical tasks are scarcely trained, especially complex ones like prescribing or ward rounds [14-16]. According to a survey by Morcke et al, residents feel best prepared for skills like history taking and physical examination after graduation, while there are more concerns about pharmacotherapy, treatments and personal aspects of professionalism [17]. This corresponds with a survey by Heaton on 2413 medical students and recent graduates in the UK, who stated that they learned pharmacotherapy mostly as “opportunistic learning during clinical attachments”. The majority (74 %) felt that their training in clinical pharmacology and therapeutics was too little or far too little [18].

Educators name workload as the most important reason for inadequacies in teaching [19]. In a survey by Delva et al., a heavy workload was associated with disorganized surface-learning for residents and clerks [20]. Interestingly, most published data on the learning conditions for final-year students have so far been based on surveys, focus-group analyses or non-structured student interviews [8]. These reports thus reflect subjective opinions rather than an objective measurement of the extent to which doctors’ workloads arising from patient care really hinder their teaching activities on ward.

The aim of our study was therefore to assess ward doctors’ workload arising from patient care in an objective way, using an individual workload index derived from our electronic hospital information system. We subsequently analyzed whether the workload arising from patient care had an impact on the supervision of medical students and students’ ward activity profiles – especially the performance of medical activities other than history taking and physical examination - during their internships in Internal Medicine.

## **Methods**

### **Study design**

Using a prospective study design, we assessed the influence of residents’ workloads arising from patient care on supervision and students’ activity profiles (medical versus non-medical activities). A medical activity was defined as an activity that could not be delegated to nurses and non-medical staff, while non-medical activities do not necessarily have to be performed by a physician. While the non-medical activities are also important and sometimes difficult and demanding, they can be performed by staff other than physicians and can thus also be learned and supervised by staff other than physicians. Our study concentrated mainly on the activities that are usually exclusively performed by physicians and thus can only be learned from and supervised by physicians.

Final-year students recorded their daily activities in a diary. The recorded activity profile and students’ supervision by the ward residents on each day were subsequently matched with a corresponding ward-specific workload index calculated from the electronic hospital information system.

### **Internship**

In our hospital the wards are run by one to three residents with one attending doctor supervising the ward round once or twice a week and discussing problems on the ward once a day. Usually, training and supervision of final year student is almost exclusively performed by the residents. Every student is obliged to attend a 16-week internship in Internal Medicine, Surgery and an elective.

### **Participants**

On the first day of their internship in Internal Medicine, 54 final-year students were asked to complete protocols of their daily activities for a period of three weeks (15 working days) using a diary (see Table 1). Students from each medical ward at our University Hospital participated. Diaries were completed between March and December 2009. We sent the students weekly reminders to fill in the diary per email. For completed diaries the students Workload Index received a voucher for a bookshop.

**Table 1:** Students diary

									2 = assisted						
	3 = performed under supervision								4 = performed without						
	or with feedback								supervision						
Day Activity	1	2	3	4	5	6	7	8	9	10	11	12	13	14	15
Administration of infusions															
Arrangement/cancelling appointments for patients															
Arterial punctures															
Documentation															
Drawing blood															
ECG interpretation															
Enquiring about results															
History taking															
Informative/explanatory consultations															
Insertion of IV lines															
Performance of errands															
Physical examination															
Prescriptions															
Presentation of patients on ward rounds															
Punctures of joints, pleura, paracentesis, etc.															
Sorting files, copying															
Transportation of patients															
Ward rounds															
Writing discharge letters															
Other activities:															
Comments:															
Please note your satisfaction with your internship: □ very high □ high □ low □ very low.															

Students completed a diary of their activities over a 15-day period and stated for specific activities whether they had (1) observed, (2) assisted, (3) performed them under supervision or (4) performed them without supervision. When the student recorded a medical activity with (3), it was counted as supervision. We then counted how many medical activities were recorded as (3) or (4) (actively performed medical activity) and how many non-medical activities were recorded with (3) or (4) (actively performed non-medical activity).

**Figure 1:**
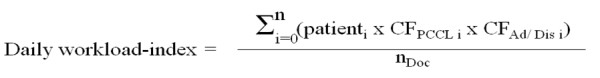
**Formula of the workload-index.** Formula for the daily workload index: n = total number of patients on the corresponding ward; CF_PPCL_ = correction factor according to the patient comorbidity complexity level (PCCL) in the national reimbursement system, _CFAd/Dis_ = correction factor according to whether the respective patient was admitted or discharged on the day in question; n_Doc_ = number of residents on that ward that day.

Informal interviews with the residents on ward (n = 18) revealed that residents considered their workload to primarily correlate with the number of patients seen, the complexity of their comorbidities, and the numbers of admissions and discharges per day. For each day recorded in the diary, we calculated a workload index (Figure 1) based on our electronic hospital information system. The index comprised the following elements:

n: The number of patients on the respective ward on the day in question.

CF_PCCL_: A correction factor for the patient comorbidity complexity level (PCCL). The PCCL modifies reimbursement for each patient in the national DRG (Diagnosis-Related Group) system according to the individual comorbidities and clinical complications arising during the corresponding hospital stay. After students diaries had been handed back, we asked residents on the wards (n = 18) to state how much more care a patient with a PCCL factor of 4 (maximum) requires compared to a patient with a PCCL factor of 0 (minimum, no relevant comorbidities). The mean result was a factor of 4.8 ± 1.3 (mean ± SD). We therefore applied a correction factor (CF_PCCL_) of 1 for PCCL 0, 1.95 for PCCL 1, 2.9 for PCCL 2, 3.85 for PCCL 3, and 4.8 for PCCL 4.

CF_Ad/Dis_: A correction factor for admission or discharge of the corresponding patient. According to the mean estimate of interviewed ward residents, admitting a patient is approximately 4.1 (± .9) times more time consuming than simply caring for the patient on ward. Discharges were estimated to be approximately 2.7 (± 1.1) times more time consuming. Therefore the correction factor CF was set to 4.1 for a patient admitted and 2.7 for a patient discharged from the ward at the corresponding day.

The overall resulting workload was divided by the number of residents present on the ward on each day examined (see Figure 1).

Workload values showed different ranges (11 to 109 points) on each of the wards under examination. For each individual ward, we therefore categorized each day according to the tertile distribution of values into high, average, or low workload.

### **Student supervision and activity survey**

Students were asked to complete diaries for a three-week period and to reflect on each day whether they had

(1) only watched,

(2) assisted the ward resident,

(3) performed the activity themselves under supervision of the ward resident, or

(4) performed the activity without supervision. (see Table 1).

The diary was constructed after an in-depth discussion with former final-year-students according to the tasks frequently performed on our medical wards. At the end there was space to fill in additional activities.

We regarded the following activities as medical (at our hospital not delegable to nurses or non-medical staff): arterial puncture, informative/explanatory consultations, documentation in patient files, ECG interpretation, prescriptions, presenting patients on ward rounds, punctures (joint, pleura, paracentesis, etc.), ward rounds, and writing discharge letters. The administration of infusions, arranging/cancelling patient appointments, taking blood samples, enquiring about test results, running errands, the administration of intravenous catheters, sorting/copying files, and patient transportation were all considered to represent non-medical activities since in our hospital they can be delegated to nurses and other staff. This distinction (medical, non-medical) was made since we wanted to assess the influence of the residents’ workload on students’ medical tasks supervision. Activities that can be delegated to nurses or other staff can be learned from and supervised by the staff in question.

History taking and physical examinations were analyzed separately, since these tasks are usually seen as core activities for medical students and thus the probability that the students practice these activities was considered to be rather high.

Every medical activity that was noted as (3) (performed under supervision) was counted as supervised activity. The students were asked to count constructive feedback as supervision, delivered either during or after the activity. We then recorded, how many medical and non-medical activities were reported as actively performed [(3) or (4)] and counted them as actively performed medical versus non-medical activities, respectively.

In addition, the students were asked to judge the motivation for teaching of the ward.

### **Ethical issues**

The study protocol was approved by the local ethics committee. Since it was not possible to ensure complete anonymity of the diaries (we needed the ward and the time period of diary completion for calculation of the workload index, see above), we were obliged to ensure that diaries were handed in on a strictly voluntary basis.

### **Statistical analysis**

A linear mixed model was used to evaluate the influence of time and workload on supervision or the amount of medical activities per day and the influence of time and supervision on the amount of medical activities per day, respectively. Due to the fact that workload and supervision varied each day, these were modeled separately as time-varying factors. Days 1–8 and Days 9–15 were analyzed individually, since the number of medical activities performed by students was only found to increase during the first eight days, with levels remaining constant across the rest of recorded ward time (see the Results section). As the residuals violated the assumption of a normal distribution, results were recalculated using the sandwich estimator [21-23]. A comparison of the results demonstrated the robustness of the applied model against deviation from a normal distribution.

The significance level was corrected for three different tests according to Bonferroni. Therefore a p-value <0.017 was regarded as being significant. The software used was JMP 9.1.3 (SAS Inc, Cary, NC, USA).

## **Results**

Student characteristics and average values for reported medical and non-medical activities are presented in Table 2.

**Table 2:** Characteristics of the participating students

Age (years)	27 ± 3
Gender (m/f)	12 m, 23 f
Motivation for this internship	8 very high25 high3 low
Actively performed non-medical activities per day	3.6 ± 1.7
Actively performed medical activities per day	1.8 ±1.6
Supervised medical activities per day	1.2 ± 1.3

Characteristics and mean activity scores (± SD) of the study cohort.

Overall, 35 of the 54 students returned diaries (65 %). Three students were excluded from analyses due to incompleteness of the data sets which were necessary to calculate the workload index. For the remaining 32 diaries (59 %), we were able to match residents` day-to-day workload index with students’ corresponding ward activities, resulting in a total of 442 working days.

At least one resident who was rated as being motivated or very motivated to teach was present on 92 % of the reported ward days. A total of 24 students reported being rather satisfied with their internship and 8 rather unsatisfied.

History taking was performed on 69 ±32 % of the recorded days, and physical examination on 74 ±30 % of the recorded days. History taking (physical examination) was supervised in 6 (5) % of recorded days.

**Figure 2:**
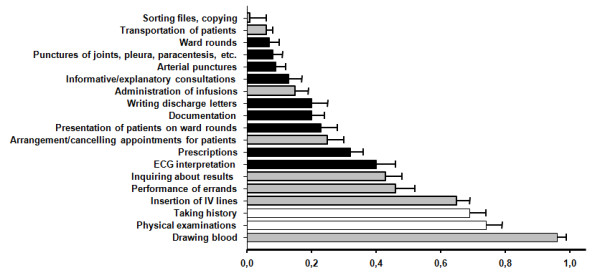
**Activities performed by the students per day**. Activities per day that the students reported to have actively performed, either with or without supervision. Gray: non-medical activities, black: medical activities. White: physical examination and history taking.

Students’diaries reported the performance of 1.8 ±1.6 medical activities other than history taking and physical examination per day. In addition, an average of 3.6 ± 1.7 non-medical activities were performed per day. In general, 1.2 ± 1.3 daily students’ activities were reported as being supervised. For every student activity that was supervised by a ward doctor, the number of actively performed medical activities increased by an average of 0.77 per day (p < 0.0001). This effect did not change over time. Figure 2 shows the distribution of the activities per day that the students reported to have actively performed.

**Figure 3:**
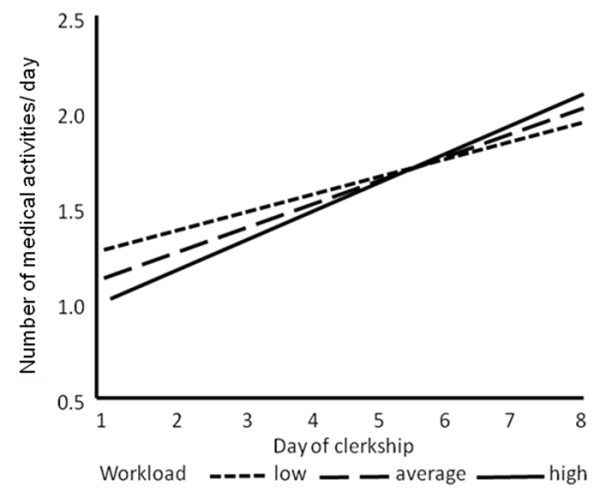
**Medical activities on day 1–8**. Trajectories of number of medical activities performed according to workload over Days 1 to 8.

The number of medical activities actively performed by students increased by an average of 0.14 per day (p < 0.0001) over Days 1–8 and by an average of 0.02 per day (p = 0.84) over Days 9–15. Modelling workload as a time-varying factor revealed that the higher the workload, the lower the initial number of medical activities (p = 0.29) and the higher the rate of subsequent increase in medial activities (p = 0.25). However, the absolute differences were rather small and remained non-significant (see Figure 3).

Between Days 9 and 15, the number of medical activities performed changed by 0.02 per day for each level of workload (p = 0.84)

Summarizing our findings, a relevant influence of the day-to-day variability of residents’ workload on the number of medical activities performed by students was not found. In addition, no relevant association was observed between increases in the applied ward workload index and an increase in non-medical activities or a decrease in student supervision (data not shown, p > .5 all).

## **Discussion**

Internships represent an essential part of medical education in most medical schools worldwide. Ideally, students in internships should practice most activities performed by a doctor under close supervision. However, it would not seem justified to rely on students practicing skills as intended, especially complex skills such as ward rounds and prescribing [14-16]. History taking and physical examination is usually covered in medical school, and the chances are high that students practice these skills during clerkships and internships. This fact has been confirmed in our study, and the low supervision rates also correspond with other studies [13]. Nonetheless, some medical activities with a higher degree of complexity are rarely taught in separate classes; it is generally assumed that such skills are acquired during internships, which in most curricula become completed during the final year of medical education [7]. In our study, we assessed whether these medical activities were practiced on the ward and whether practice ideally occurred under the supervision of the ward doctor.

Interestingly, while at least one resident who was rated as being “motivated or very motivated to teach” was present on the ward on 407 of the 442 examined days, supervision was reported to have been scarce. However, there was a clear association between the degree of reported supervision and the number of medical activities. We were not able to detect a significant association of measured workload and the number of reported supervised medical activities or student activity profiles. Thus, in order to increase supervision, reducing the workload arising from patient care alone might not be sufficient, and additional factors as e.g. the individual learner-supervisor-relationship or structured feedback training may be necessary [9].

A total of 24 students reported being rather satisfied with their internship and 8 rather unsatisfied. We therefore assume that our data are not overly distorted by dissatisfaction.

A limitation of our study is that the students primarily engaged in non-medical activities, which might explain why we failed to find any changes in activity profiles on days with a high workload. The number of reported medical activities only increased over the first eight days of the internship. Students may be surmised to have acquired the role of a clinical helper rather than a learner over this period of time. This may imply that we failed to detect a potentially negative influence of doctors’ workload on students’ supervision and activity profiles. Another explanation of this negative finding might be workload yet very high at baseline, which would impede or even extinguish any amount of teaching activity regardless of further workload increases. In addition, some parts of the workload can be postponed to the other day. While the work for admissions is usually completed the same day, discharge letters can be prepared in advance or on subsequent days. In addition we investigated only the workload arising purely from patient care calculated from the electronic hospital information system. But some workload also stems from research activities, other teaching activities, conferences and so on. The effect of the subjectively perceived workload on supervision might be an interesting research question for future research.

In order to ensure that high-quality medical education is provided despite increasing workload, several measures are conceivable, including e.g. the implementation of protected time for supervision and teaching on the ward or training complex activities in special classes or simulated environments in order to increase students’ confidence in practicing these activities on ward [24,25].

## **Conclusions**

While there was a clear association between supervision and number of medical activities performed by the final year medical students, no association of the workload on the degree of supervision could be detected. Thus, in order to increase supervision, reducing the workload arising from patient care alone might not be sufficient.

## **Competing interests**

The authors declare that they have no competing interest.

## **Authors’ contributions**

NC conceived of the study and drafted the manuscript. RT participated in the study design and helped drafting the manuscript. CE performed the statistics and helped drafting the manuscript. FH participated in the study design and the interviews with the ward residents. RR participated in the study design and the interviews with the ward residents. PW participated in the study design, the interviews with the former final year students and the data acquisition and helped drafting the manuscript. All authors read and approved of the final manuscript.

## Pre-publication history

The pre-publication history for this paper can be accessed here:

http://www.biomedcentral.com/1472-6920/12/24/prepub
